# The effect of curcumin-piperine on cardiometabolic, inflammatory and oxidative stress factors and macular vascular density in optical coherence tomography angiography (OCTA) in patients with non-proliferative diabetic retinopathy: Study protocol for a randomized, double-blind controlled trial

**DOI:** 10.22038/AJP.2022.21512

**Published:** 2023

**Authors:** Sepide Amini, Amirhossein Sahebkar, Alireza Dehghani, Bijan Iraj, Abdolreza Rezaeian-Ramsheh, Gholamreza Askari, Muhammed Majeed, Mohammad Bagherniya

**Affiliations:** 1 *Nutrition and Food Security Research Center, Department of Community Nutrition, School of Nutrition and Food Science, Isfahan University of Medical Sciences, Isfahan, Iran *; 2 *Department of Biotechnology, School of Pharmacy, Mashhad University of Medical Sciences, Mashhad, Iran*; 3 *Ophthalmology Ward, Feiz Hospital, Isfahan University of Medical Sciences, Isfahan, Iran*; 4 *Isfahan Endocrine and Metabolism Research Center, Isfahan University of Medical Sciences, Isfahan, Iran*; 5 *Sabinsa Corporation, East Windsor, NJ, United States*

**Keywords:** Diabetes mellitus, Curcumin-Piperine, Diabetic retinopathy, OCTA

## Abstract

**Objective:**

Curcumin is a safe phytochemical with antioxidant, anti-inflammatory, antidiabetic, and lipid-lowering effects. This study aims to investigate the efficacy of curcumin-piperine in non-proliferative diabetic retinopathy.

**Materials and Methods:**

In this double-blind randomized trial, 60 diabetic retinopathy patients after meeting the inclusion criteria will be randomly assigned to two groups of curcumin-piperine supplementation (1000 mg per day for 12 weeks) or receiving placebo. The density of small blood vessels in the retina by optical coherence tomography angiography (OCTA), fasting blood glucose, triglyceride, renal indices (blood urea nitrogen and creatinine), high-sensitivity C-reactive protein, total antioxidant capacity, total oxidant status, body mass index, waist circumference, and weight will be measured.

**Conclusion:**

If the beneficial effects of curcumin on diabetic retinopathy are observed, this safe, this natural and inexpensive herbal supplement can be considered a therapeutic solution in these patients.

## Introduction

Diabetes can cause various microvascular complications, especially diabetic retinopathy (DR) (Behl et al., 2016). The number of people with diabetes is expected to increase from about 382 million in 2013 to 592 million in 2035, so, the risk of developing diabetic retinopathy will increase in the coming decades (Guariguata et al., 2014; Nanditha et al., 2016). DR greatly affects the quality of life and interferes with daily activities (Simó-Servat et al., 2019). Most cases of blindness and visual impairment in working-age adults are due to DR (Yoon et al., 2006). DR has two distinct levels, including the early stage of non-proliferative diabetic retinopathy (NPDR) and the advanced stage of proliferative diabetic retinopathy (PDR) (Mohamed et al., 2007). NPDR is characterized by microaneurysms, retinal hemorrhages, and microvascular abnormalities, and PDR is associated with the pathological pre-retinal formation of new vessels (neovascularization and angiogenesis) (Stitt et al., 2016). The resulting vessels have thin and fragile walls, and the risk of bleeding from these vessels is high; these conditions can lead to sudden and complete blindness (Brownlee, 2001). 

Hyperglycemia is one of the major risk factors for DR. Exposure of retinal capillary endothelial cells to high glucose levels stimulates the formation of free radicals (oxidative stress) (Behl et al., 2016). Controlling blood sugar and systemic blood pressure reduces the risk of microvascular complications of diabetes (Behl et al., 2016). Inflammation and oxidative stress also play an important role in diabetic patients (Lontchi-Yimagou et al., 2013; Karam et al., 2017). 

Current therapies for DR include the use of intravitreal drug agents, laser photocoagulation, and vitreous surgery, all of which focus on the management of microvascular complications (Gonzalez et al., 2016). Vascular endothelial growth factor (VEGF) causes angiogenesis by affecting the vessel wall, thus, playing a vital role in the pathogenesis of DR (Behl et al., 2016). At present, intravitreal anti-VEGF drugs are the primary treatment in various stages of DR (Yoon et al., 2006). Although they improve vision in many retinal diseases, side effects are common (Nguyen et al., 2012; Ip et al., 2008). One of the side effects of anti-VEGF injection is intraocular inflammation (Tolentino, 2011). Moreover, its high cost has placed a significant burden on the healthcare system (Choi et al., 2013). Another method is laser therapy which may be associated with retinal damage (David et al., 2012). Given these concerns, finding practical, effective, inexpensive, and affordable treatments with minimal side effects is essential.

Plants containing polyphenols have traditionally been used as antidiabetic agents (Watal et al., 2014; Srinivasan, 2005). Among these, plants that have the most negligible side effects are receiving more attention (Nabavi et al., 2015). Curcumin, the biologically active substance in turmeric, is a low molecular weight hydrophobic flavonoid with numerous health benefits including antioxidant and anti-inflammatory activities (Jeenger et al., 2015; Neerati et al., 2014; Mohajeri et al., 2020; Parsamanesh et al., 2018; Farhood et al., 2019; Gorabi et al., 2019; Mortezaee et al., 2019; Shakeri et al., 2019). No significant adverse effects are associated with supplementation with curcumin, even at doses above 8 g/day (Mirzaei et al., 2017). Many regulatory proteins, including chemokines, interleukins, hematopoietic growth factors, and transcription factors, are modulated by curcumin, thus reducing the inflammatory process (Peddada et al., 2019). In several studies, curcumin has been shown to downregulate tumor necrosis factor (TNF) -α (Li et al., 2013) and C-reactive protein (Adibian et al., 2019).

Curcumin supplementation improved glycemic factors, low-density lipoprotein, very low-density lipoprotein, and triglycerides in patients with diabetes (Neerati et al., 2014). It also significantly lowered blood glucose levels (Nabavi et al., 2015) and modulated retinal disorders in diabetic rats by preventing retinal thinning, apoptosis of retinal ganglion cells and inner nuclear layer cells, and thickening the retinal capillary basement membrane (Yang et al., 2018). In obese mice, curcumin consumption significantly improved glycemic status (blood glucose, glucose tolerance, and, HbA1C) and insulin sensitivity (Weisberg et al., 2008). 

The effectiveness of curcumin on diabetes has been discussed in several recent meta-analyses. In a way that curcumin improves blood sugar, and lipid factors, and reduce insulin resistance, it has a beneficial effect on improving and preventing the progression of diabetes (Altobelli et al., 2021; Zhang et al., 2021; Poolsup et al., 2019). In a trial study, the efficacy of Meriva (each tablet containing 500 mg of Meriva equivalent to 100 mg of curcumin) was evaluated in patients with DR. The results of this study showed an improvement in arterial vascular response and peripheral edema score (Steigerwalt et al., 2012).

Curcumin has low bioavailability due to its low solubility in aqueous solvents and instability at physiological pH which limit its use in clinics (Anand et al., 2007). Interestingly, the combination of curcumin with piperine (an alkaloid derived from the plant *Piper nigrum* L.) has better gastrointestinal absorption and reduces curcumin's systemic excretion. Piperine increases the bioavailability of curcumin by binding to the enzyme glucuronidase in the intestine, preventing glucuronidation and reducing the excretion of curcumin from the stool (Kaur, 2012).

Optical Coherence Tomography Angiography (OCTA) is a new noninvasive, colorless eye volume imaging technique (Matsunaga et al., 2014) used to evaluate common eye diseases such as acute macular degeneration, DR, arterial and venous occlusion, and glaucoma (Carlo et al., 2015). Optical coherence tomography (OCT) has been used since 1991 as a suitable fraternal imaging tool to analyze eye disorders. However, it is not able to provide sufficient and appropriate information about retinal and choroidal vasculature (Fingler et al., 2007), while OCTA can display structural and functional data (including blood flow) simultaneously (Schwartz et al., 2014). Compared to other eye imaging modalities, including FA (Fluorescein angiography) and ICGA (indocyanine green angiography), OCTA has many benefits. For example, it is noninvasive, achieves good volume scans in a matter of seconds, uses motion contrast instead of intravenous dye, and provides more accurate information (Matsunaga et al., 2014).

To our knowledge, no study assessed the effect of curcumin supplementation on macular vascular density in OCTA in patients with DR. Thus, this study aims to investigate the efficacy of curcumin-piperine co-supplementation on cardiometabolic, inflammatory, and oxidative stress factors and macular vascular density in OCTA in patients with non-proliferative diabetic retinopathy. 

## Materials and Methods

**Trial design**

This is a randomized, placebo-controlled, double-blind, parallel-arm clinical trial performed in university hospitals affiliated of Isfahan University of Medical Sciences, Isfahan, Iran. Recruitment has started in February 2022.

Sixty patients in the age group of 30 and 65 years old, diagnosed with DR with the approval of an ophthalmologist, imaging, or CT scan will be enrolled (According to Figure 1). Patients will be randomly assigned in a 1:1 ratio into two groups: 1) Placebo capsules, each containing 505 mg of maltodextrin, two capsules/day, 2) Curcumin-piperine capsules, each containing 500 mg of curcumin and 5 mg of piperine, two capsules/day. Patients will be asked to consume capsules after breakfast and dinner for 12 weeks.

**Figure:**
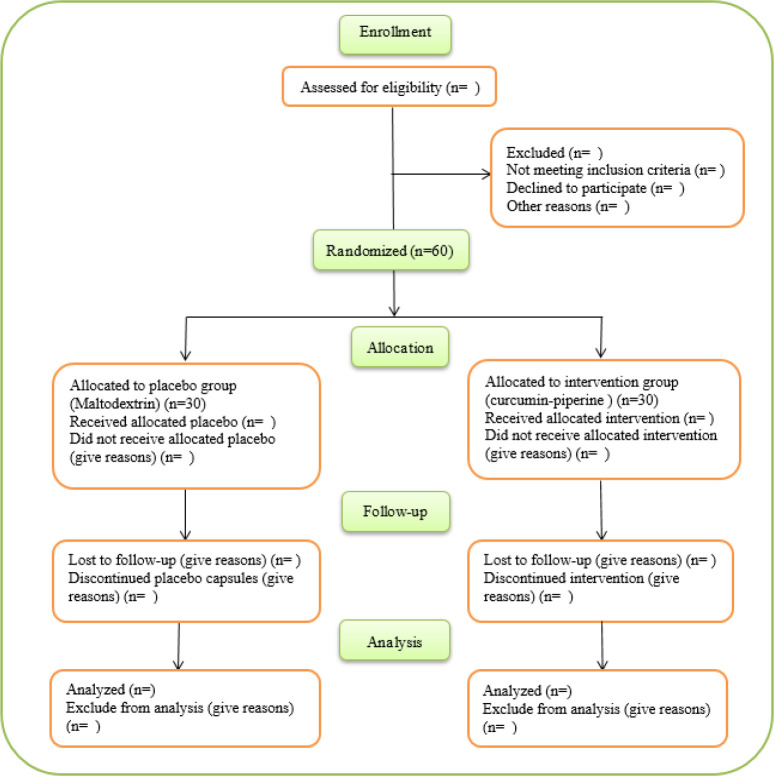
Participant flow diagram according to the Consolidated Standards of Reporting Trials (CONSORT) 2010 statement

**Participants**

**Inclusion criteria**

Inclusion criteria are 30 to 65 years of age, diagnosis of DR with the approval of an ophthalmologist, imaging, CT scan or PCR, and definitive diagnosis of diabetes by a diabetic physician (fasting blood glucose above 126 mg/dl, measured twice, or HbA1C greater than or equal to 6.5%).

**Exclusion criteria**

Exclusion criteria are allergies to herbal products such as turmeric and pepper, following a special diet in the last 3 months, taking anticoagulants such as heparin, warfarin, or aspirin, insulin consumption, pregnancy or lactation, taking medications or herbal supplements in the last 3 months, current use of anti-VEGF, use of treatments including laser therapy, eye surgery, and intraocular injections, glaucoma, macular edema, and ocular uveitis, or having certain diseases such as congenital diseases, type 1 diabetes, immunodeficiency, cancer, or uncontrolled diabetes. Patient will also be excluded if they are dissatisfied to continue the intervention, taking less than 80% of curcumin piperine supplement, reporting any adverse events after taking the supplement, or if other treatments of the patient, including treatment of hypertension, hyperlipidemia, etc. change during the treatment period. 

**Randomization and interventions**

**Figure 2:**
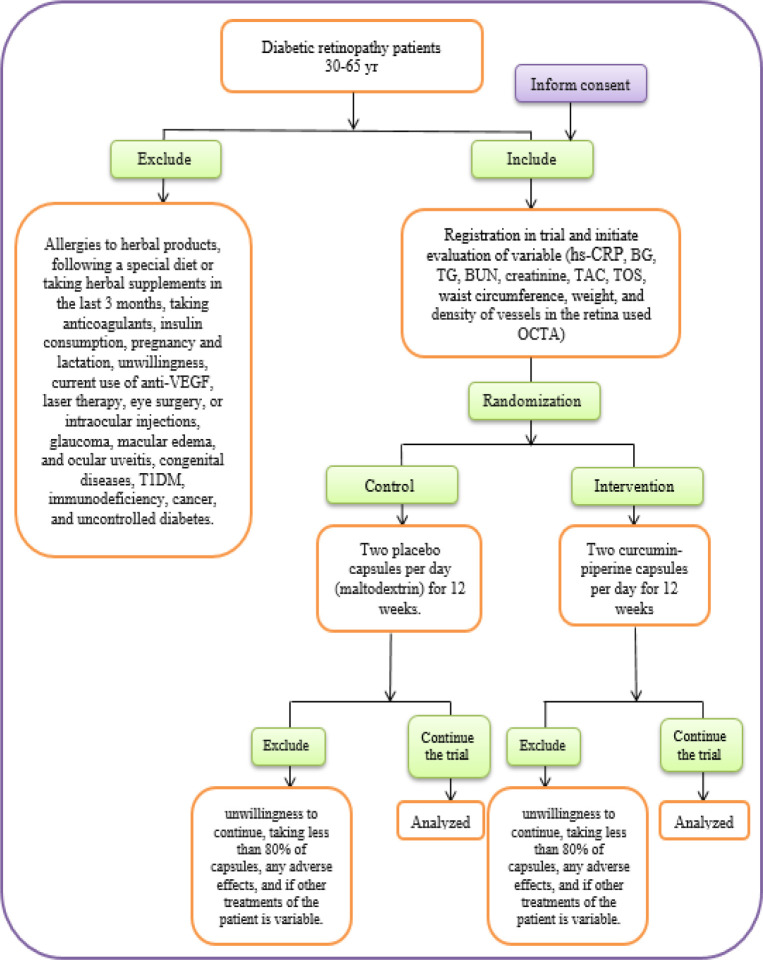
Trial procedure flow sheet

Participants will be randomized using four blocks based on age and gender in 1:1 ratio into two groups using computer generated randomization (https://www.sealedenvelope.com/simple-randomiser/v1/lists) (According to Figure 2(. Allocation concealment will be performed by sequentially numbered containers.

Group 1 consists of 30 patients who will be received two curcumin-piperine capsules per day for 12 weeks (each capsule contains 500 mg curcumin and 5 mg piperine; a total of 1000 mg curcumin and 10 mg piperine per day). 

Group 2 will be the control and consists of 30 patients who will be received two placebo capsules per day (maltodextrin) (each capsule contains 505 mg maltodextrin; a total of 1010 mg of maltodextrin per day) for 12 weeks. 

Study compliance will be evaluated by giving a supplement box to the participants every month. At the end of every month, the number of unused supplements will be counted and recorded. Patients will be reminded to take supplements weekly by phone and SMS during the study. Participants will be excluded from the study if they take less than 80% of the supplements during the study.

If a patient misses a dose of the supplement, it is recommended that they take the supplement immediately after the reminder. If much time has elapsed since the allotted time for consumption one dose will be considered missing. 

**Blinding**

This study will be conducted in a double-blind context, so capsules (curcumin and placebo) will be labeled A and B by the company in similar packages. Capsules will be similar in terms of shape, size, color, and odor. Investigators, participants, laboratory staff, outcome assessors, and data analyzers will be blinded to treatment assignment until final analyses will be performed.

**Measurements and outcomes**

Demographic variables (including age, sex, marital status, smoking, medical history and history, level of education, occupation, supplementation, and medication) will be collected from all participants by completing a general information questionnaire at the beginning of the trial. Anthropometric indices (waist circumference, weight, and BMI) will be measured at the beginning and at the end of the study. Bodyweight will be measured using a digital scale (Seca scale, Germany) at approximately 0.1 kg in the morning on an empty stomach without shoes and with minimal clothing. Waist circumference and height will be measured by an inelastic meter with an accuracy of 0.1 cm.

Fasting blood samples (10 ml) will be collected at baseline and 12 weeks after intervention at 8:00 am to check blood factors. high-sensitivity C-reactive protein (hs-CRP), blood glucose, triglyceride, and renal indices (blood urea nitrogen and creatinine) using the ELISA kit, also, oxidative stress indices including total antioxidant capacity (TAC) and total oxidant status (TOS) using the colorimetric method by commercial Kiazist kits (Tehran, Iran) will be measured at the beginning of the study and repeated 12 weeks after the intervention in groups A and B (Table 1).

**Table 1:** Time line and applied tests. BMI: Body mass index, WC: Waist circumference, OCTA: Optical coherence tomography angiography, hs-CRP: high-sensitivity C-reactive protein, TG: Triglyceride, BUN: Blood urea nitrogen, TAC: Total antioxidant capacity, TOS: total oxidant status

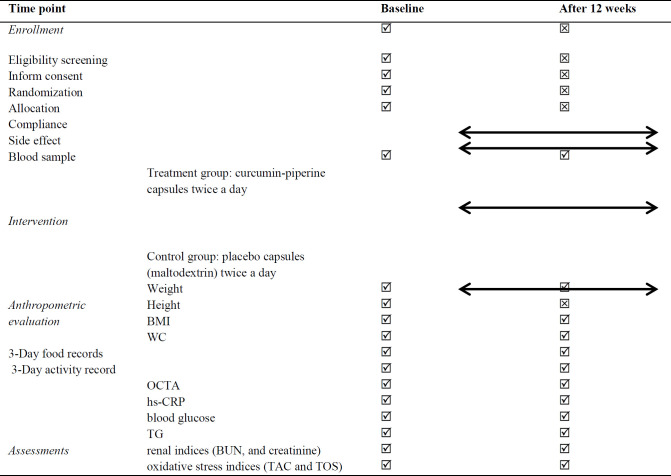

**OCTA evaluation**

The density of small blood vessels in the retina will be measured by the new, noninvasive, and useful imaging method, OCTA (optical coherence tomography angiography), by an ophthalmologist before and after the study. This imaging method uses Ophthalmic Optical Coherence Tomography System (OPTOVUE) (Germany serial number 32319R2).

OCTA is able to provide clear and relevant information about volumetric blood flow quickly. In this method, by comparing the difference in intensity or amplitude of multiple signals between consecutive scans, the movement of red blood cells in the retinal blood vessels can be observed (Kim et al., 2013; Choi et al., 2013). OCTA has a higher field of view and better image quality compared to conventional OCT. It also takes less time to scan (Schwartz et al., 2014; Spaide et al., 2015).

**Sample size**

The most important finding of this study is the effects of curcumin on the accumulation of small blood vessels in the eye area (based on OCTA findings). Due to the novelty of this evaluation method, according to the researcher's knowledge, there is no similar study in this regard. Therefore, the sample size is calculated based on fasting blood glucose in a previous study (Seyyedebrahimi et al., 2018) as follows: 

Based on 80% power, an α level of 0.05, and a potential dropout rate of 10%, 60 participants (i.e. 30 participants in each group) would be required. The formula n=2[(Z1-α/2+Z1-β)2×S2]/ Δ2=2[(1.96+0.84)^2^× (27)^2^]/ (25)^2^ was used to calculate the sample size.

**Safety**

In previous studies, no significant risk was observed during supplementation with curcumin, even at doses above 8 g/day (Mirzaei et al., 2017). However, if any adverse side effects are reported after taking, the supplements will be stopped immediately.

**Ethics approval**

The scientific code of this clinical trial is 3400358, the ethical code is IR.MUI.RESEARCH.REC.1400.253, and the IRCT code is IRCT20201129049534N5. Written consent will be received from all patients participating in the study. 

**Statistical methods**

The present study will report quantitative variables as average (standard deviation) and qualitative variables as number (percentage). The normality of the distribution of quantitative variables will be assessed using the skewness index and the Q-Q plot diagram. Intragroup analyses will be performed using paired t-test and intergroup analyses using independent t-test and ANCOVA. The distribution of qualitative variables will be compared between the two groups using the chi-square test. SPSS software version 22 will be used to analyze the data with a significance level of p<0.05. The analysis will eventually be performed in the form of Intention-To-Treat (ITT) and Per-Protocol (PP).

## Discussion

Curcumin is the main biologically active substance in turmeric (Jeenger et al., 2015) which is clinically safe even at doses higher than 8 g per day (Mirzaei et al., 2017). Anti-inflammatory, antioxidant, and lipid-lowering effects (Neerati et al., 2014; Li et al., 2013), as well as improved glycemic status (blood glucose, glucose tolerance, and HbA1C) and insulin sensitivity, have been observed after curcumin (Nishiyama et al., 2005; Weisberg et al., 2008). Although curcumin is rapidly metabolized and has low bioavailability due to low solubility in aqueous solvents and instability at physiological pH (Anand et al., 2007), its bioavailability is increased in the presence of piperine (Kaur, 2012).

DR is one of the most important complications of diabetes that severely affects the quality of life and causes most blindness and visual impairment cases in working-age adults (Yoon et al., 2006). The use of anti-inflammatory and antioxidant, sugar-lowering, and fat-reducing drugs in the final prognosis of the disease is very effective (Chaturvedi et al., 2001; Stitt et al., 2016). 

Curcumin has been reported to reduce the levels of IL-6 (Kondamudi et al., 2015; Chen et al., 2015), TNF-α (Li et al., 2013), and C-reactive protein in earlier studies (Adibian et al., 2019). It also has anti-inflammatory effects mediated by increasing the regulation of peroxisome proliferator-activated receptor-γ (PPAR-γ) (Jacob et al., 2007). Curcumin modulates oxidative conditions by direct reaction with reactive oxygen species (ROS), reduction of hydroxyl radicals (∙OH) (Barzegar and Moosavi-Movahedi, 2011), and peroxynitrite (ONOO^−^) (Kim et al., 2013). In an animal study, curcumin reduced angiogenesis by reducing VEGF expression in the retinas of diabetic mice and prevented retinal weakening (Yang et al., 2018). Curcumin can regulate the activity of insulin receptors and improve insulin sensitivity. It has also been reported to effectively regulate blood sugar in diabetes (Moselhy et al., 2011; Peeyush et al., 2009).

Clinical studies have shown that curcumin increases the levels of high-density lipoprotein (HDL-C) and decreases the levels of low-density lipoprotein (LDL-C), triglycerides, total cholesterol, and non-HDL-C cholesterol (Yang et al., 2014; Panahi et al., 2016). It also inhibits lipid peroxidation (Pivari et al., 2019; Mokgalaboni et al., 2021). 

**Figure 3:**
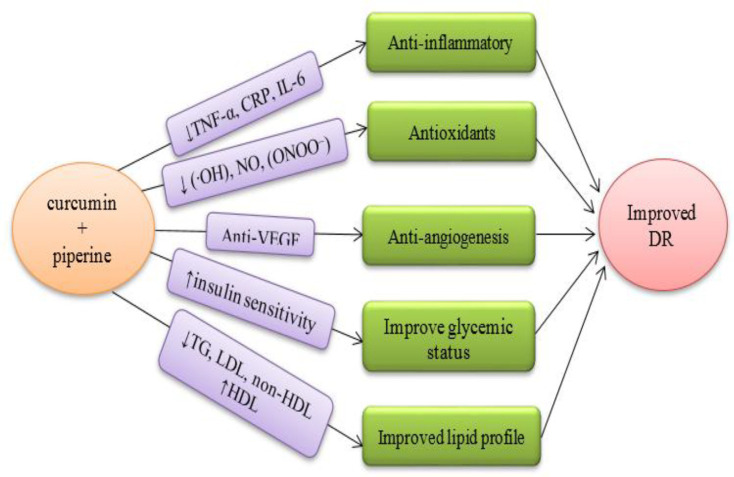
Possible effects of curcumin on the improvement of diabetic retinopathy. (curcumin-piperine can improve diabetic retinopathy by improving inflammatory factors, reducing free radicals, anti-VEGF effects, increasing insulin sensitivity, and improving lipid factors)

Many systematic review and meta-analyses have suggested the effectiveness of curcumin on diabetes. In these studies, the effect of curcumin on improving blood sugar, increasing insulin sensitivity and improving lipid factors have been emphasized (Mahdavi et al., 2021; Zhang et al., 2021; Altobelli et al., 2021; Poolsup et al., 2019). Therefore, according to Figure 3, it seems that patients with DR may be candidates for the herbal supplement curcumin-piperine. To the best of our knowledge, this is the first study to investigate the effect of curcumin-piperine supplementation as a specific nutritional and supportive factor on macular vascular density in OCTA in patients with DR.

The most important strength of our protocol is that if significant benefits emerge, as this treatment requires very low costs and displays minimal side effects, it can be easily used clinically, and adherence will be high. The main limitation of our protocol is that the intervention time is relatively short. In addition, due to ethical issues, we will not be able to study the effect of curcumin-piperine alone. Also, due to economic constraints, it is impossible to measure important factors such as TNF-α, IL-6, and HbA1C. Another limitation is that due to lack of funding, it is not possible for us to consider a blood biomarker of the curcumin to assess adherence to supplements.

Due to the lack of medical care and pharmacological agents for DR, if this trial shows beneficial effects of curcumin on patients with diabetic retinopathy, this dietary supplement would be considered as a safe, natural and inexpensive supplement with minimal side effects for this condition. In addition to this study, in future, more well-designed clinical trials will be needed to clarify the effects of curcumin on DR and to determine the optimum dose and type of curcumin for the intervention.

## Conflicts of interest

Muhammed Majeed is the founder of Sami-Sabinsa group of companies. The other authors have nothing to disclose.
